# Stroke volume variation guided fluid therapy in septic shock with ARDS

**DOI:** 10.1186/cc10840

**Published:** 2012-03-20

**Authors:** S Jog, D Patel, M Patel, S Sable

**Affiliations:** 1 Deenanath Mangeshkar Hospital and Research Centre, Pune, India

## Introduction

Optimal fluid resuscitation guided by central venous pressure (CVP) in patients having septic shock with ARDS is a perplexed issue having risk of underfilling and worsening of shock versus fluid overload leading to pulmonary edema. Whether stroke volume variation (SVV) (Flotrac-Vigileo system) guided fluid resuscitation has an impact on improvement of shock, oxygenation and mortality were tested in this single-center prospective study [1,2].

## Methods

Inclusion criteria were: (1) septic shock patients with dose of norepinephrine ≥0.1 μg/kg/minute or dopamine ≥10 μg/kg/minute; (2) CVP ≥12 mmHg; (3) PO_2_/FiO_2 _ratio ≤200 with ARDSnet protocol ventilation under deep sedation. Exclusion criteria were atrial/ventricular arrhythmias, spontaneous triggering of inspiration, established renal failure needing continuous renal replacement therapy (CRRT). During the 24-hour study period, SVV was continuously monitored with the third-generation Flotrac-Vigileo system (version 3.02). Intravenous fluids were given in the boluses of 250 to 500 cm^3 ^to keep SVV <12% throughout the study period. Vasopressor infusion was titrated to keep MAP >70 mmHg.

## Results

Thirty-seven patients with severe sepsis-induced multiorgan dysfunction syndrome with average APACHE II score of 24.6 and PEEP of 8.2 cm were enrolled. SVV guided fluids received during the 24-hour study period were 5.1 ± 2.6 l. Arterial lactates reduced significantly without worsening of hypoxia. The PO_2_/FiO_2 _ratio increased significantly at 24 hours. Twenty-two out of 37 survived (59.45%) until hospital discharge. See Table 1.

**Table 1:** Hemodynamic variables at 0 and 24 hours

Variable	0 hours	24 hours	*P *value
CVP	15.51	16.2	>0.1
MAP	73.6	76.4	>0.1
SVV%	13.61	10.8	>0.1
Arterial lactate	3.9 ± 2.6	2.3 ± 1.5	<0.001
PO_2_/FiO_2_	120.6 ± 42	193.4 ± 76	<0.001

## Conclusion

SVV guided fluid therapy in septic shock with ARDS may improve shock by optimizing preload in a targeted way without worsening oxygenation.
